# From Proteomic Analysis to Potential Therapeutic Targets: Functional Profile of Two Lung Cancer Cell Lines, A549 and SW900, Widely Studied in Pre-Clinical Research

**DOI:** 10.1371/journal.pone.0165973

**Published:** 2016-11-04

**Authors:** Luís Korrodi-Gregório, Vanessa Soto-Cerrato, Rui Vitorino, Margarida Fardilha, Ricardo Pérez-Tomás

**Affiliations:** 1 Cancer Cell Biology Research Group, Department of Pathology and Experimental Therapeutics, Faculty of Medicine, University of Barcelona, Campus Bellvitge, Feixa Llarga s/n, 08907, L'Hospitalet de Llobregat, Spain; 2 Laboratory of Signal Transduction, Department of Medical Sciences, Institute for Research in Biomedicine—iBiMED, Health Sciences Program, University of Aveiro, Campus de Santiago, 3810–193, Aveiro, Portugal; 3 Department of Medical Sciences, Institute for Research in Biomedicine—iBiMED, Health Sciences Program, University of Aveiro, Campus de Santiago, 3810–193, Aveiro, Portugal; 4 Department of Physiology and Cardiothoracic Surgery, Faculty of Medicine, University of Porto, Alameda Professor Hernâni Monteiro, 4200–319, Porto, Portugal; University of South Alabama Mitchell Cancer Institute, UNITED STATES

## Abstract

Lung cancer is a serious health problem and the leading cause of cancer death worldwide. The standard use of cell lines as *in vitro* pre-clinical models to study the molecular mechanisms that drive tumorigenesis and access drug sensitivity/effectiveness is of undisputable importance. Label-free mass spectrometry and bioinformatics were employed to study the proteomic profiles of two representative lung cancer cell lines and to unravel the specific biological processes. Adenocarcinoma A549 cells were enriched in proteins related to cellular respiration, ubiquitination, apoptosis and response to drug/hypoxia/oxidative stress. In turn, squamous carcinoma SW900 cells were enriched in proteins related to translation, apoptosis, response to inorganic/organic substances and cytoskeleton organization. Several proteins with differential expression were related to cancer transformation, tumor resistance, proliferation, migration, invasion and metastasis. Combined analysis of proteome and interactome data highlighted key proteins and suggested that adenocarcinoma might be more prone to PI3K/Akt/mTOR and topoisomerase IIα inhibitors, and squamous carcinoma to Ck2 inhibitors. Moreover, ILF3 overexpression in adenocarcinoma, and PCNA and NEDD8 in squamous carcinoma shows them as promising candidates for therapeutic purposes. This study highlights the functional proteomic differences of two main subtypes of lung cancer models and hints several targeted therapies that might assist in this type of cancer.

## Introduction

Cancer is a heterogeneous group of diseases that results from abnormal, autonomous and uncontrolled cell growth and differentiation, promoting tumor formation and metastasis. Tumors are commonly characterized by six hallmarks: insensitivity to anti-growth signals, evasion of apoptosis, self-sufficiency in growth signals, sustained angiogenesis, limitless replicative potential and tissue invasion and metastasis[1]. Moreover, there are two novel emerging hallmarks: deregulation of the cellular energetics and avoidance of immune destruction[2]. Signaling cascades, which usually control cellular homeostasis, are deregulated in tumorigenesis through genetic, epigenetic and somatic alterations[3]. Hence, the acquisition of these hallmarks is facilitated by an enabling characteristic of cancer cells: genomic instability[2]. Lung cancer is the world leading cause of cancer-related mortality in both genders. The 2012 estimated rates of the European Cancer Observatory (ECO), states that lung cancer contributed with one fifth of the total cancer-related deaths[4]. The main causes of lung cancer include tobacco smoke (direct or indirectly, account for more than 85%), asbestos, ionizing radiation (e.g. radon) and other air pollutants. Conversely, only 10% of smokers will develop lung cancer and not all exposed to the other environmental factors will develop it[5], highlighting the importance of intrinsic factors. At the histological level, lung cancer is divided into two major types: small-cell (SCLC) and non-small-cell lung carcinoma (NSCLC). SCLC accounts for around 12–15% of all cases, being however more aggressive and metastatic than NSCLC[6]. NSCLC is less aggressive and spreads more slowly but is more common, accounting for at least 85–88% of all lung cancer cases. NSCLC can be further divided into three subtypes: adenocarcinoma (50%), squamous cell carcinoma (30%), and large cell carcinoma (10%)[6]. Human cancer-derived cell lines provide to research an almost unlimited and self-replicating source of tumoral cells. The human lung adenocarcinoma cell line A549 was established by D.J. Giard back in 1972 through an explant culture of a carcinomatous tissue from a 48 year-old Caucasian male[7] and deposited in ATCC cell line bank (CCL-185^TM^) by M. Lieber[8]. The A549 cells are characterized as a hypotriploid human alveolar basal epithelial cells and are widely used as an *in vitro* model for type II pulmonary epithelial cells as well as a model of lung adenocarcinoma[8]. These cells grow adherently in monolayer and are suitable as a transfection host. The SW900 cells (HTB-59^TM^) also grow adherently in monolayer and are hypotriploid epithelial cells. The cell line was established in 1975 by A. Leibovitz through a biopsy tissue of a grade IV squamous carcinoma from a 53 year-old Caucasian male[9] and is a cell line commonly used as a squamous carcinoma model[10, 11]. Inactivation of the tumor suppressor CDKN2A gene locus (homozygous, c.1_471del471/p.M1_*157del) is present in both cell lines[12, 13]. The CDKN2A gene produces three different transcripts: *p16INK4α*, *p14ARF* and *p12*. While the specific function of the last is less known, the other two transcripts have important tumor suppressor functions. The p16INK4α protein causes cell cycle arrest in G1 phase due to the inhibition of the cyclin-dependent kinases CDK4/6, thereby inhibiting the phosphorylation of the retinoblastoma protein. The p14ARF protein induces cell cycle arrest in G1 and G2 phases by interacting with MDM2 and preventing the p53 degradation[12]. On the contrary, restauration of transcripts, particularly *p16INK4α* in the A549 cell line, leads to suppression of cell growth and enhanced sensitivity to cisplatinum, the first-line treatment for many lung cancers[12]. Both cell lines also harbor an activating mutation in Rat Sarcoma (RAS) pro-oncogene K-Ras protein (SW900 is heterozygous for c.35G>T/p.G12V and A549 is homozygous for c.34G>A/p.G12S) that belongs to the small GTPase superfamily[14]. The Ras/Raf/MEK/ERK pathway can be activated through EGFR, FGFR and PDGFR tyrosine kinase receptors and is important in the control of cellular proliferation, differentiation, survival and stemness[15]. The NSCLC displays high frequency of K-Ras mutations that is usually associated with tobacco smoking. Interestingly, the mutations in Ras proteins appear to be mutually exclusive from other mutations in components of the signaling pathway (e.g. EGFR mutations)[15]. A combined therapy of EGFR (gefitinib, AstraZeneca) and MEK1 (AZD6244, AstraZeneca) inhibitors prevents cell growth in the A549 cell line whereas single treatment with these inhibitors has little effect[14]. Another study has reported that a combined therapy of a MEK inhibitor (CI-1040, Pfizer Inc.) and a mTOR inhibitor (rapamycin/sirolimus) had no addictive or synergistic effect but the A549 cell line was sensitive to both inhibitors in separate[16]. The A549 cell line is in turn wild-type against other common mutations in lung cancer such as, EGFR, PIK3CA, TP53, ALK and PTEN. The SW900 cell line is also wild-type for these mutations with the exception for the inactivated mutation in the tumor suppressor gene TP53 (homozygous, c.499C>T) that plays an important role in regulating the DNA damage response[17]. Modernly, ‘-omics’ analyses have been developed to define ‘fingerprints’ in cancer cells and to study drug effects. These approaches allow the measurement of transcript and protein expression levels and protein modifications. Since in signaling cascades, proteins and their modifications play a central role, proteomics is a powerful instrument in the discovery of novel biomarkers. The direct analysis of tumor cells proteome offers information that cannot be acquired by the study of genetics and epigenetics. Many studies have demonstrated the power of mass spectrometry (MS)-based proteomic approaches to identify altered proteins as potential lung cancer biomarkers[18–20]. For comparative protein quantification using MS, several methods use stable isotope labelling; however, the use of label-free approaches as an alternative methodology has recently emerged. Besides, there is substantial evidence that label-free methods provide higher dynamic range of quantification[21, 22]. In the present work, by using label-free MS, the protein expression of these two lung cancer cell lines, adenocarcinoma (A549) and squamous carcinoma (SW900), was studied and the differences from the two major subtypes of lung cancer are presented. To our knowledge, this is the first time that a proteomic comparison between the two most frequent lung cancer subtypes has been performed. These results will be useful for a better understanding of the cell biology of the main subtypes of lung cancer and can be used in future studies to develop targeted antitumoral therapies.

## Material and Methods

### Cell Culture

Human lung adenocarcinoma epithelial cells (A549; ATCC^®^ CCL-185^TM^) were cultured in Dulbecco's Modified Eagle's Medium (DMEM, Biological Industries, Beit Haemek, Israel) and squamous carcinoma epithelial cells (SW900; ATCC^®^ HTB-59^TM^) in RPMI 1640 medium (Biological Industries). Both media were supplemented with 10% heat-inactivated fetal bovine serum (FBS; Life Technologies, Carlsbad, CA), 100 U/ml penicillin, 100 μg/ml streptomycin, and 2 mM L-glutamine all from Biological Industries. Cells were grown at 37°C under a 5% CO_2_ atmosphere.

### Sample Preparation

Low-passage number mycoplasma-free cells were seeded at a concentration of 10^5^ cells/mL and allowed to grow for 48 h. Before reaching confluence, cells were harvested using a scraper and washed twice with 1X PBS. Cells were then lysed in a buffer containing 1% Triton X-100, 20 mM MOPS, 1 mM DTT, 5 mM EDTA, 2 mM EGTA supplemented with phosphatase (20 mM sodium fluoride, 20 mM sodium pyrophosphate, 60 mM beta-glycerophosphate and 1 mM sodium orthovanadate) and protease (Roche cOmplete mini cocktail, Roche Diagnostics and 5 μM pepstatin A) inhibitors for 15 min on ice. The lysate was sonicated three times for 5 sec and centrifuged at 16,100 g for 15 min at 4°C. Supernatants were kept and protein concentrations were determined using standard BCA assay (Pierce, Rockford, IL, USA). Three independent replicates for each cell line were processed.

### 1D-Gel Electrophoresis

Samples were precipitated (100 μg) in 20% trichloroacetic acid (TCA) by incubation for 30 min at 4°C. After centrifugation at 4°C, 14,000 g, for 15 min, the pellets were washed twice in ice-cold acetone, dried at room temperature and resuspended in 1X Laemmli buffer (10% glycerol, 62.5 mM Tris-HCl pH 6.8, 2% sodium dodecyl sulfate, 0.5% β-mercaptoethanol and bromophenol blue). Samples were resolved on a 4–20% SDS-PAGE gradient gel and stained with coomassie blue colloidal G250 to visualize the gel bands.

### Tryptic In-Gel Digestion

For tryptic in-gel digestion, each lane was cut in 16 pieces, and the pieces were digested with trypsin to be identified by matrix assisted laser desorption ionization-time of flight (MALDI-TOF/TOF). The gel pieces were washed three times with 50 mM ammonium bicarbonate for 20 min and 50% acetonitrile (ACN) for 15 min, to neutralize and remove the staining. The gel pieces were then washed one time with ACN for 10 min and dried in a SpeedVac (Thermo Savant, Holbrook, NY, USA). Sequence grade modified porcine trypsin in 50 mM ammonium bicarbonate was then added (19 μL of 10 μg/mL, Promega, Madison, WI, USA) to the dried gel pieces and allowed to digest for 1 h at 37°C. Finally, 25 μL of 25 mM ammonium bicarbonate were added and the gel pieces allowed to incubate overnight at 37°C.

### Peptide Identification by LC-MS/MS

Trypsin digestion was stopped by the addition of 10% formic acid (FA) and 30 min incubation at room temperature. Tryptic peptides were then extracted by the addition of 10% FA/50% ACN and lyophilized in a SpeedVac. After that, tryptic peptides were resuspended in solubilization solution (13 μL of 50% ACN/0.1% FA). All peptide mixtures were analyzed twice. The tryptic digests were then separated using an Ultimate 3000 (Dionex, Sunnyvale, CA, USA) onto a 150 mm × 75 μm Pepmap100 capillary analytical C18 column with 3 μm particle size (Dionex/LCa Packings) at a flow rate of 300 nL/min. The gradient started at 10 min and ramped to 50% buffer B (85% ACN, 0.04% trifluoroacetic acid) over a period of 45 min. The chromatographic separation was monitored at 214 nm using an ultraviolet detector (Dionex/LC Packings) equipped with a 3 nL flow cell. The peptides eluting from the column were mixed with a continuous flow of matrix solution (270 nL/min, 2 mg/mL alpha-Cyano-4-hydroxycinnamic acid in 70% ACN/0.3% trifluoroacetic acid and internal standard Glu-Fib at 15 fmol) in a fraction microcollector (Probot; Dionex/LC Packings) and directly deposited onto the liquid chromatography-MALDI plates. Samples were analyzed using a 4800 MALDI-TOF/TOF Analyzer (Ab SCIEX, Concord, Ontario, Canada). A signal/noise threshold of 50 was used to select peaks for MS/MS analyses. Data from all slices was merged into one file and submitted to Mascot search (Mascot software, v.2.1.0.4; Matrix Science Ltd) for peptide/protein identification. Searches were performed against the SwissProt protein database (March 2013) for *Homo sapiens*. A MS tolerance of 30 ppm was found for precursor ions and 0.3 Da for fragment ions, as well as two missed cleavages and methionine oxidation as variable modification. The confidence levels accepted for positive protein identification were above 95%. A minimal Mascot peptide score of 30 was determined by a reverse database search, which revealed a false positive rate below 5% for identified proteins. Furthermore, proteins identified with 1 peptide were manually validated when MS/MS spectra presented at least 4 successive amino acids covered by *b* or *y* fragmentations. The exponentially modified protein abundance index (emPAI) was used to obtain an estimation of the absolute protein amounts using the number of sequenced peptides per protein obtained from Mascot search as it is stated in [23].

### Bioinformatics Analysis

Venn diagrams were generated using the online tool Venny v2.1[24]. The Pearson coefficients were calculated using the GraphPad Prism software v5.0 (GraphPad Software Inc, SanDiego, CA, UA). The database for annotation, visualization and integrated discovery (DAVID) v6.7 was used to retrieve the gene ontology (GO) terms (biological processes, cellular components and molecular functions) of the A549 and SW900-specific proteins. A gene threshold of 3 and a p-value of 0.05 were selected to obtain the term lists. The terms within the lists were subsequently grouped using the functional annotation clustering tool of DAVID using the following parameters: initial and final group membership of 2 and a similarity threshold of 0.30–0.45 depending on the GOs being analyzed. The human integrated protein-protein interaction reference (HIPPIE, vSep 05, 2014) database was used to retrieve the protein-protein interactions (PPIs) from the protein list obtained from MS. A filter of 0.63 or 0.73 were applied that represents the interactions with medium and high confidence score (second and third quartile of the HIPPIE score distribution, respectively). The resulting output list of HIPPIE interactors was also passed through a context filter using a homemade database of genes expressed in lung tissue and/or lung cell lines. The homemade database was retrieved from past cell lines studies[25–29] and from well-established tissue expression databases: The Human Protein Atlas (THPA v13[30]), VeryGene (v1.9[31]), C-It[32], TiGER (v1.0[33]) and BioGPS[34]. From the tissue expression databases, only high confidence expressions (well-supported) where retrieved. The HIPPIE interactors present in at least two databases or studies, were selected as lung tissue/cell line positives and their respective PPIs with the MS list of proteins retained. SteinerNet webserver was used to reveal hidden components in A549 and SW900 networks by integrating the proteomics and the interactome data[35]. The Cytoscape platform (v3.2.1[36]) for network visualization and analysis (NetworkAnalyzer, v2.7[37]) was used to build both the final and the SteinerNet networks of A549 and SW900 cell lines. The GOs and pathways of altered (fold-regulation > 2) and specific proteins for each cell line were retrieved using the Cytoscape plugin ClueGO (v2.1.7[38]), which evaluates the enrichment of the main GO categories, including cellular components, biological processes, and molecular functions using a right-sided hypergeometric distribution and False Discovery Rate (FDR). In order to determine significantly over-represented GO terms (molecular function), the terms with a FDR < 0.05 were considered as Kappa significant values. Genes classified as significantly overrepresented were validated by the Benjamini & Hochberg method. GO Term fusion that retains the most representative parent or child term in parent-child relationship was used. GO term grouping, that associate terms in functional groups, was applied using the kappa score. GO terms were represented as nodes in the final network and proteins present in each GO node were also denoted as nodes using color discrete mapping (red for down-regulated > -2 and green for up-regulated >2).

### Western Blot

Four independent replicates for adenocarcinoma and squamous carcinoma cell lines were prepared using standard denaturing conditions. In brief, both cell lines were seeded (10^5^ cells/mL) and allowed to grow for 48 h. Total protein extracts were obtained from cells by the addition of lysis buffer (85 mM Tris-HCl pH 6.8 and 2% SDS) supplemented with protease inhibitors (1 mM phenylmethylsulfonyl fluoride and serine and cysteine protease inhibitor Roche cocktail). Protein concentration was determined by BCA protein assay (Pierce, Rockford, IL, USA) using bovine serum albumin (BSA) as a standard. After that, 40 μg of protein extracts were separated by 12% SDS-polyacrylamide gel electrophoresis and transferred to Immobilon-P membranes (Millipore, Bedford, MA, USA). Membranes were blocked in 5% dry milk or 5% BSA, both diluted in 1X TBS–Tween (50 mM Tris–HCl pH 7.5, 150 mM NaCl and 0.1% Tween 20) for 1 h and then incubated overnight with primary antibodies, according to the manufacturer’s instructions. Primary antibodies rabbit anti-vimentin (Cat#3932, 1:1000) and rabbit anti-topoisomerase IIα (Cat#4733, 1:1000) were acquired from Cell Signaling (Cell Signaling Technology, Beverly, MA, USA). Primary antibodies rabbit anti-cytokeratin 18 (Cat#CSB-PA10629A0Rb, 1:2500), rabbit anti-annexin A4 (Cat#CSB-PA001845ESR2HU, 1:2500), rabbit anti-calvasculin/S100A4 (Cat#CSB-PA020632HA01HU, 1:500) and rabbit anti-galectin-1 (Cat#CSB-PA012882HA01HU, 1:500) were acquired from Cusabio (Cusabio Biotech, Wuhan, China). Primary antibodies goat anti-actin (Cat#, 1:400), mouse anti-porin/VDAC1 (Cat#ab14734, 1:1000), rabbit anti-filamin-B (Cat#AB9276, 1:1000) and rabbit anti-EGFR (Cat#E-2760, 1:3000) were acquired from Santa Cruz Biotechnology (Santa Cruz Biotechnology, Santa Cruz, CA, USA), Abcam (Abcam, Cambridge, MA, USA), Chemicon International (Chemicon International, Temecula, CA, USA) and Sigma-Aldrich (Sigma-Aldrich, St Louis, MO, USA), respectively. Primary antibody binding was detected with secondary IgG-HRP antibodies goat anti-rabbit (Cat#sc-2004, 1:1000), goat anti-mouse (Cat#sc-2005, 1:1000) or donkey anti-goat (Cat#sc-2020, 1:1000) all from Santa Cruz Biotechnology, followed by chemiluminescence reaction using an ECL detection kit (Amersham, Buckinghamshire, UK). Actin was used as loading control. Images were captured on an Image Quant LAS 500 (GE Healthcare, Little Chalfont, UK) and band densitometries were retrieved using the Image Studio Lite software (v5.0, Li-COR, Lincoln, NE, USA).

### Statistics

Expression levels of the altered proteins were first normalized using an actin loading control and then averaged. A one-way analysis of variance (ANOVA) was then was employed to test for the statistical significance of the obtained values between both cell lines using the Statgraphics Centurion software (v.16.1.11, StatPoint Technologies Inc., Warranton, VA, USA).

## Results and Discussion

### Proteomic Profile of the Lung Cancer Lines

**Fig 1:**
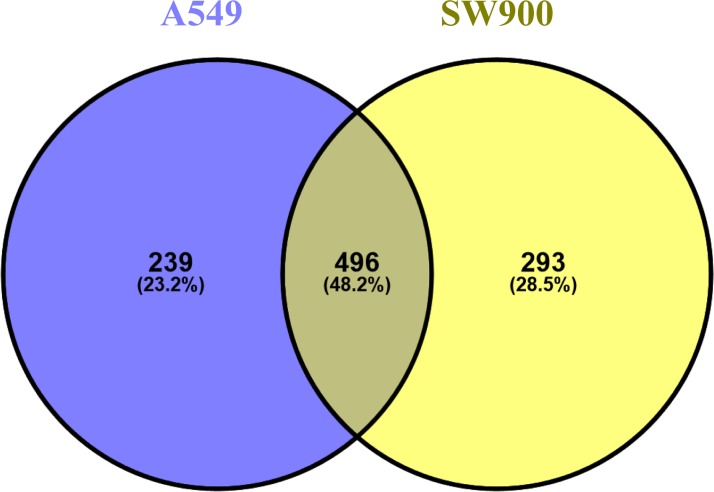
Global proteome analysis of the lung cancer cell lines. Venn diagram highlighting the distribution of the identified proteins per cell line in numbers and in percentage, evidencing the overlapped and unique proteins (Venny 2.0.2).

The proteomic profiles of human lung adenocarcinoma (A549) and squamous carcinoma (SW900) cell lines, representative of the most incident lung cancers, were studied by MS. A total of 735 different proteins in A549 and 789 in SW900 were obtained. When comparing both cell lines, 496 proteins are shared, 239 are A549-specific and 293 are SW900-specific (Fig 1 and S1 Table). The calculated Pearson coefficients of the replicates evidence high similarity between the obtained proteomic profiles (0.88 for both cell lines). The dynamic range profile obtained for both cell lines is 11.6. Analysis of the protein differential expression (fold-change > 2) between cell lines (A549 vs SW900, S1 Table) showed that 68 proteins are overexpressed and 83 proteins are underexpressed. The distribution of the protein ratios is near-to-normal with 255 out of 496 proteins (51.4%) being present in similar amounts (S1 Fig). The most altered proteins (fold-change > 4) are depicted in Table 1. Of special mention is keratin 18 (KRT18) that is overexpressed in A549 (4.65 fold-change). This keratin is an intermediate filament cytokeratin that is commonly associated with simple epithelium and is highly abundant in lung adenocarcinoma when comparing with squamous carcinoma[39, 40]. The KRT18 has been shown to be involved in resistance to tumor necrosis factor (TNF) induced cell death and to be highly expressed in paclitaxel resistant A549-Taxol cell line[27, 41]. The most highly overexpressed protein in A549 (13.58 fold-change) was annexin A4 (ANXA4), a member of the calcium-dependent phospholipid binding annexin family that are involved in cancer invasion and metastasis[42]. More particularly, annexin A4 is implicated in paclitaxel drug resistance in A549 and in platinum resistance in several cancers[43, 44]. Also related to annexins is calgizzarin (S100A11, 6.23 fold-change), a member of the EF-hand-type Ca^2+^-binding proteins S100 family. S100 proteins and annexins are involved in plasma membrane repair and it has already been shown that S100A11 is overexpress in several cancers (e.g. lung and colon) and it is associated with metastasis and a poor prognosis[45]. The Rab small GTPase oncogenes, RAB11A and RAB5C, which are important players in integrin trafficking and cell migration and proliferation, are also overexpressed in A549 (4.27 fold-change for both)[46]. Also related, SLC3A2 (CD98, 9.73 fold-change) is an oncogenic protein commonly highly expressed on the surface of tumor cells and its interaction with β1 integrins is important in cellular transformation and growth[47]. The mitochondrial proteins VDAC1, COX5 and NDUFS3 were also found to be overexpressed in A549. Several cytoskeletal related proteins were overexpressed in SW900 (> 4 fold-change), namely emerin (EMD), stathmin (STMN1), vimentin (VIM), myosin 9 (MYH9) and myosin regulatory light chain 12 alpha (MYL12A). Emerin is a type II inner nuclear envelope structural protein that connects the nuclear lamina to the actin cytoskeleton being important for nuclear formation such as the well-known lamins A and C. Loss of nuclear envelope proteins like lamins and possibly emerin are common in cancer cells and might be involved with nuclear envelope morphological aberrations and aneuploidy[48]. Another inner nuclear matrix protein overexpressed in SW900 (4.09 fold-change) was matrin 3 (MATR3), whose function is largely unknown but could be implicated in transcription by stabilizing several mRNAs[49]. Stathmin, which was overexpressed in SW900 (4.56 fold-change), is an important regulatory protein of microtubule dynamics and involved in cell cycle progression and motility. Overexpression of stathmin was associated with a poor prognosis in patients with NSCLC and a knockdown of this protein decreased cellular proliferation and invasion[50]. Moreover, stathmin expression has also been correlated with poor prognosis in patients treated with both platinum and paclitaxel chemotherapeutic drugs[51]. Another cytoskeletal protein highly overexpressed in SW900 was vimentin (8.03 fold-change). Vimentin is a type III intermediate filament protein that has a role in tumor initiation and progression, including tumorigenesis, epithelial-to-mesenchymal transition and metastasis[52]. Calcyclin (S100A6), another member of the S100 family, was found to promote cancer progression through cell survival and apoptotic routes and in our study was overexpressed in the SW900 cell line (4.26 fold-change)[53]. Galectin 1 (LGALS1), is a glycoprotein that has been shown to be overexpressed in many tumors including lung cancer where its inhibition reduces metastasis through the induction of integrin α6β4 and Notch1/Jagged2 signaling pathway[54]. Moreover, it was also shown to induce tumor-mediated immune anergy through the IL-10 signaling pathway, tumor progression and chemoresistance[55]. In our study galectin 1 was the second highest overexpressed protein in SW900 (5.91 fold-change) when comparing with the A549 cell line. Moreover, the SW900 overexpressed proteins, SLC2A1 (2.11 fold-change), TFRC (2.07 fold-change) and HSPB1 (1.71 fold-change), also showed up in a previous study comparing squamous carcinoma and adenocarcinoma patient samples using super-SILAC and label-free proteomics, being highly expressed in squamous carcinoma[40].

**Table 1:** Most differential expressed proteins (fold-change > 4) between both cell lines (A549 vs SW900).

Gene	Protein name	A549 (emPAI)	SW900 (emPAI)	Fold-change
ANXA4	Annexin A4	2.91	0.21	13.58
SLC3A2	4F2 cell-surface antigen heavy chain	0.55	0.06	9.73
COX5A	Cytochrome c oxidase subunit 5A, mitochondrial	1.53	0.23	6.63
S100A11	Protein S100-A11 (Calgizzarin)	2.05	0.33	6.23
VDAC1	Voltage-dependent anion-selective channel protein 1	0.66	0.12	5.51
NDUFS3	NADH dehydrogenase [ubiquinone] iron-sulfur protein 3, mitochondrial	0.63	0.12	5.27
KRT18	Keratin, type I cytoskeletal 18	1.80	0.39	4.65
TKT	Transketolase (TK)	0.77	0.17	4.47
RAB11A	Ras-related protein Rab-11A	0.65	0.15	4.27
RAB5C	Ras-related protein Rab-5C	0.68	0.16	4.27
FLNB	Filamin-B	0.39	0.09	4.22
PTMS	Parathymosin	1.41	0.34	4.18
				
MATR3	Matrin-3	0.14	0.57	-4.09
S100A6	Protein S100-A6 (Calcyclin)	1.32	5.62	-4.26
EMD	Emerin	0.13	0.55	-4.29
MYH9	Myosin-9	0.21	0.92	-4.35
NEDD8	Neural precursor cell expressed developmentally down-regulated protein 8	0.44	2.02	-4.55
STMN1	Stathmin (Leukemia-associated phosphoprotein p18)	0.22	1.01	-4.56
MDH2	Malate dehydrogenase, mitochondrial	0.21	1.00	-4.64
GAPDH	Glyceraldehyde-3-phosphate dehydrogenase	0.48	2.45	-5.11
MYL12A	Myosin regulatory light chain 12A	0.19	1.06	-5.54
LGALS1	Galectin-1 (Gal-1)	0.26	1.54	-5.91
VIM	Vimentin	1.12	8.97	-8.03

**Fig 2:**
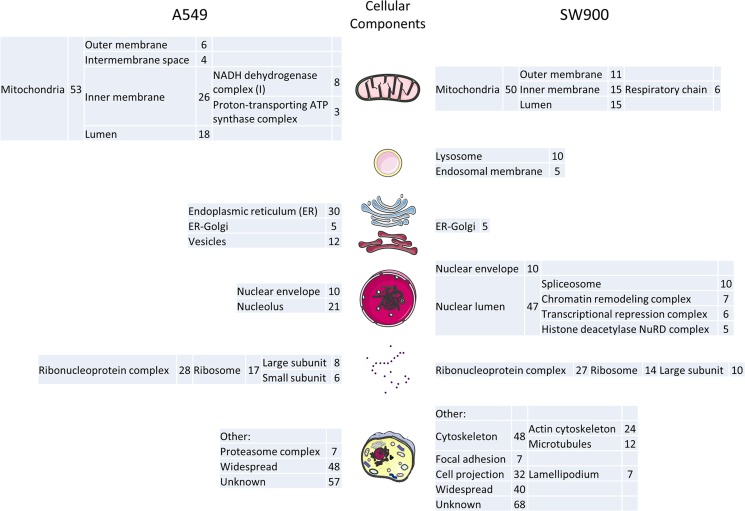
GO analysis of the specific proteins of adenocarcinoma and squamous carcinoma cell lines: cell components. Enriched GO terms were retrieved using DAVID database. For the A549 cell line 127 proteins out of 239 (53%) and for the SW900 cell line 174 proteins out of 293 (59%) were classified in the GO terms. The enrichment was performed considering a p-value of 0.05 and a minimum number of 3 genes per term. Parts of the figure were adapted from Servier Medical Art templates available at /www.servier.co.uk/content/servier-medical-art. Servier Medical Art is licensed under a Creative Commons Attribution 3.0 Unported License (http://creative-commons.org/licenses/by/3.0/).

**Fig 3:**
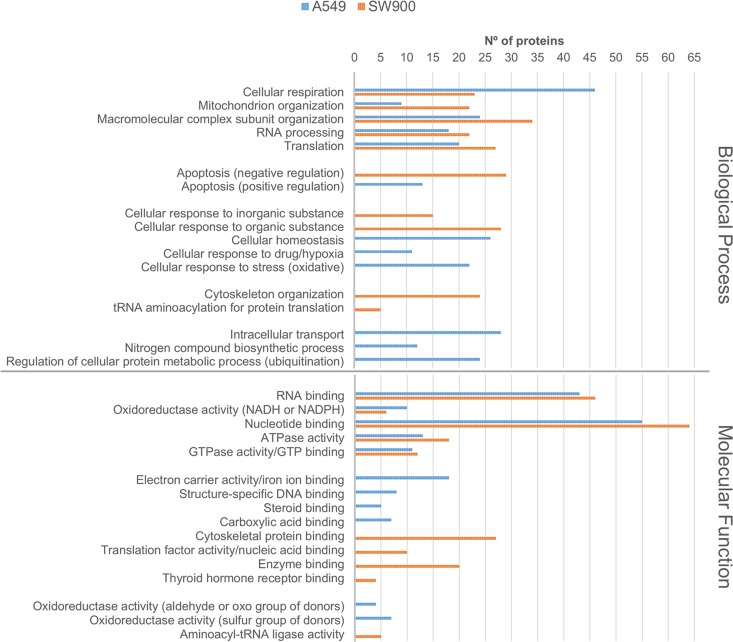
GO analysis of the specific proteins of adenocarcinoma and squamous carcinoma cell lines: biological process and molecular function. Enriched GO terms were retrieved using DAVID database. Biological process: for the A549 cell line 141 proteins out of 239 (59%) and for the SW900 cell line 152 proteins out of 293 (52%) were classified in the GO terms and clustered. Molecular function: for the A549 cell line 114 proteins out of 239 (48%) and for the SW900 cell line 136 proteins out of 293 (46%) were classified in the GO terms clustered. The clustering was performed considering a p-value of 0.05 and a minimum number of 2 terms per cluster.

**Fig 4:**
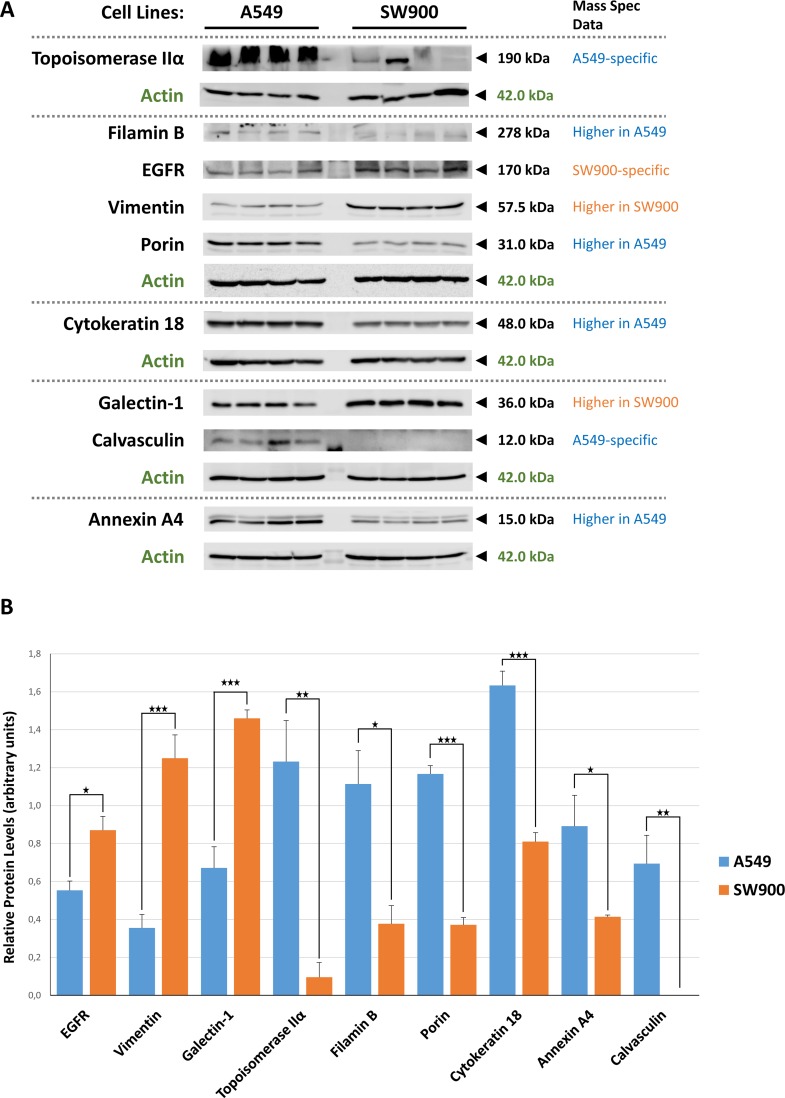
Expression levels of several altered proteins in adenocarcinoma and squamous carcinoma cell lines. Four independent extracts of A549 and SW900 cell lines were prepared and analyzed using the Western blot technique for expression comparison purposes. **(A)** Western blot images of the four replicates in both cell lines. Actin was used as loading control. **(B)** Protein band densitometries were obtained, values were normalized using the internal actin control and finally averaged. In the graph (Mean ± SE, n = 4), * *p* < 0.05, ** *p* < 0.01 and *** *p* < 0.001, indicate significant changes between the analyzed cell lines following one-way ANOVA.

Regarding the cell line specific proteins, the GOs terms related to each protein (cellular component, biological process and molecular function) were analyzed and clustered whenever required using the DAVID database in order to provide a glimpse of the major processes present in each cell line. In the A549 cell line it is clear the enrichment in endoplasmic reticulum, mitochondrial inner membrane, small subunit of the ribosome, nucleolus and proteasome complex related proteins comparing with the SW900 cell line (Fig 2). In turn, the SW900 cell line is enriched in lysosomal/endosomal, nuclear lumen, cytoskeletal and focal adhesion proteins. From the nuclear lumen, special mention to the chromatin remodeling complex proteins (SWI/SNF complex: SMARCD1, SMARCE1, ACTB and GTF2F1), since its master player, SMARC4/BRG1, is commonly mutated in NSCLC and promotes aggressiveness[56]. This complex is important to expose regions of DNA that will be critical to transcription, DNA replication and repair and the A549 cell line possesses and inactivating mutation in this gene (homozygous for c.2184_2206del23/p.Q729fs*4)[56, 57]. The abrogation of this complex in the A549 cell line might explain why other proteins related to it where not present. These localizations explain in part the biological processes and molecular functions (Fig 3) obtained for both cell lines (S2 and S3 Tables). In A549 the biological processes of positive regulation of apoptosis (proteins localized in the ER, mitochondria inner membrane, small subunit of the ribosome and in the nucleolus: BAX, DAP3, HMOX1, RYR2, TGM2, TOP2A, TXNDC12), homeostasis, response to drug/hypoxia and oxidative stress (like the nucleolus proteins: TOP2A, HMOX1, RBM14), intracellular transport (proteins localized in the endoplasmic reticulum, Golgi and vesicles: RAB14, RAB1A, RAB2A, RAN, RYR2, SEC23B, SEC61B, TMED10, TMED2 and VAM7), nitrogen compound biosynthetic process and ubiquitination (proteasomal proteins: PSMA3, PSMC6, PSMD12, PSMD4, PSMD7, PSMD9 and RAD23B) were highlighted. Additionally, the A549-specific proteins, AGR2, TGM2 and S100A4, were previously shown to be differentially expressed in adenocarcinoma when comparing to squamous carcinoma[40]. In SW900 cell line the biological processes are focused on negative regulation of apoptosis (like the proteins localized on the nuclear lumen: ACIN1, MYO18A, SQSTM1, XRCC5 and CTNNB1), response to inorganic and organic substances, cytoskeleton organization (cytoskeletal proteins like ACTC1, ACTN1, CTNNB1, DBN1, DCTN2, DSTN, DYNLL1, FSCN1, GSN, MAP1B, MYBPC3, MYH14, MYO6 among many others) and tRNA aminoacylation for protein translation (Fig 3). Regarding the molecular function in the A549 cell line, more proteins related to the structure-specific DNA (proteins related to the nucleolus or to the proteasome complex: MCM4, SAFB, RAD23 and PIN4) and steroid/carboxylic acid binding (proteins related to the endoplasmic reticulum and linked before to cellular respiration: CAV1, P4HA2, CYP26A1 and PLOD2) were retrieved. Moreover, mitochondrial and endoplasmic reticulum proteins associated before to cellular homeostasis, response to oxidative stress and cellular respiration were also emphasized in molecular function terms such as, iron ion binding, electron carrier activity and oxidoreductase activities (e.g. GLRX5, P4HA2, CYP26A1, CYP4F2, POR, CYB5B, FDXR, NDUFS6, NDUFS1, TXNDC12, PLOD2, NDUFS8, CAT and HMOX1). On the other side, SW900 cell line has more proteins related to the binding to cytoskeletal, nucleic acid, enzyme and thyroid hormone and to the aminoacyl-tRNA ligase activity, the last one already linked before to the tRNA aminoacylation for protein translation (e.g. DARS, EPRS, KARS, VARS, YARS2) (Fig 3). In addition, the SW900-specific proteins, KRT14, FSCN1 and AHNAK2, were previously shown to be differentially expressed in squamous carcinoma when comparing to adenocarcinoma[40]. We have further corroborated by Western blot four proteins overexpressed in A549 comparing to SW900 (Filamin B/FLNB, Porin/VDAC1, Cytokeratin 18/KRT18 and Annexin A4/ANXA4) and two A549-specific proteins (Topoisomerase II/TOP2A and Calvasculin/S100A4) (Fig 4A). As expected, the A549-specific proteins were the ones with the highest differential expression with Calvasculin/S100A4 being absent under our conditions (Fig 4B). We have also corroborated the two proteins with more overexpression in SW900 comparing to A549 (Vimentin/VIM and Galectin-1/LGALS1) and one protein SW900-specific (EGFR). These results validate our workflow and give confidence to our bioinformatics analysis.

### Interactome of the Lung Cancer Lines

The specific protein-protein interactions of A549 and SW900 cell lines were retrieved in order to build interactome networks for each cell line. For that purpose, the HIPPIE database was used to recover the PPIs from the protein lists obtained from MS. HIPPIE database integrates interaction data from 10 different source databases and 11 experimental studies and provides a confidence score of the interactions. The score is calculated from the number of experimental studies that detected the interaction, the type (quality) of the used techniques and the number of other organisms in which the interaction was also validated[58]. The medium-high confidence interacting partners were retrieved and applied a home-made lung tissue/cell line filter to obtain only the context relevant ones (S4 and S5 Tables). The final networks were assembled using the Cytoscape platform for network visualization. For the sake of simplicity, only the highest score PPIs without self-loops, are presented (S2 and S3 Figs). For the A549 cell line, 538 proteins from MS have PPIs from databases and from SW900, 606 proteins. For the A549 cell line, this corresponds to a total of 2349 interactors (nodes) and 5881 PPIs included in the network and for the SW900 cell line, 2459 interactors and 6463 PPIs. Network parameter analysis using the NetworkAnalyzer tool was performed to gain insight regarding the parameters of the nodes. Important network parameters include degree (connectivity) and the betweeness centrality[59]. The degree of a node (e.g. protein) is the number of edges (connections/interactions) linked to it. Nodes with high degree are commonly referred as hubs. In PPIs networks, hub proteins are more likely to be essential than non-hub proteins[60]. In other terms, much of the regulation in a network occurs and is mediated through hub proteins. In the A549 network (S2 Fig) the proteins expressed by SUMO2 (Small ubiquitin-like modifier 2, degree 493), SUMO1 (Small ubiquitin-like modifier 1, degree 187), HDAC1 (Histone deacetylase 1, degree 139), YWHAZ (14-3-3ζ/KCIP-1, degree 136), HSP90AA1 (heat shock protein 90 kDa alpha, degree 121) and YWHAG (14-3-3γ, degree 120) are the ones that present higher degrees, and so, are hub proteins in the network. Similarly, SW900 network present the same hub proteins plus the EGFR (Epidermal growth factor receptor, degree 153) (S3 Fig). Moreover, the betweeness centrality of a node reflects the control that a node exerts over the interactions of other nodes in the network[61]. The nodes with high betweeness centrality are commonly referred as bottlenecks, and like hubs, represent important nodes in biological networks[62]. Other groups also introduced the definition of “party-hubs” and “date-hubs”, being the first, hubs with high degree but with only local importance for some modules (functions) and the second ones, hubs with high range of connections required for the global organization of the biological modules in the PPI network[63]. While the “date-hubs” seem to fit the hub-bottleneck definition, the “party-hubs” seem to be hub-nonbottleneck nodes. Additionally, nonhub–bottlenecks are generally nodes that are involved in the cross-talk of different processes[63]. In both networks, A549 and SW900, the hub proteins stated above are also the ones with more betweeness centrality and so they are also bottlenecks. This reflects their high importance in the network and in the biological processes/molecular functions of these cell lines.

### Integration of the Proteome and Interactome Data

**Fig 5:**
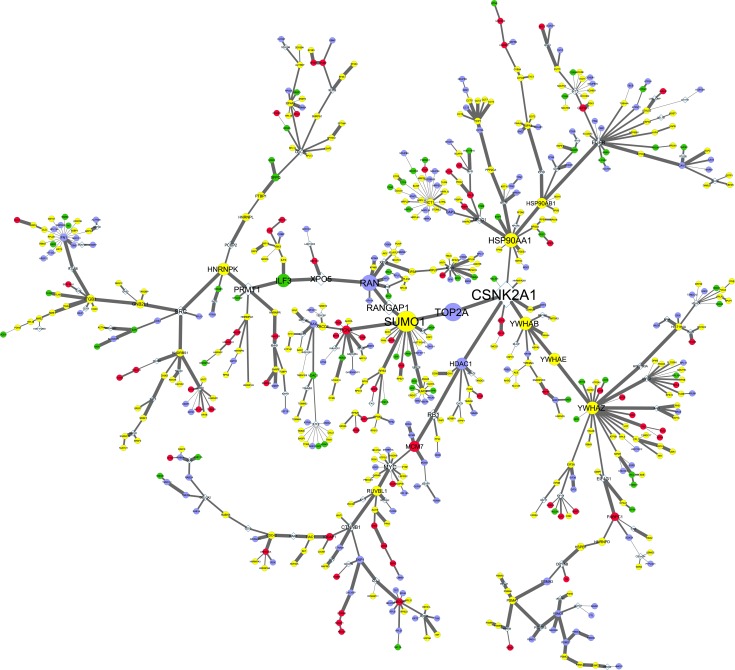
Integration of proteome and interactome data of the adenocarcinoma cell line. SteinerNet webserver was used to reveal hidden components in A549 network by integrating the proteome (MS, fold-regulation) and the interactome data (HIPPIE, interaction scores). From the original network, 175 terminal nodes were excluded (23.7%) and 563 terminal nodes included (76.3%). Circular nodes denotes proteins obtained from MS, whereas diamond nodes are proteins obtained from HIPPIE database. Node and letter size are related to the betweeness centrality (high betweeness centrality represent important nodes in the network, also called bottlenecks) of the proteins and was calculated using the Cytoscape NetworkAnalyzer tool. Edge width shows the interaction score confidence. Node color is depicted as following (A549 vs SW900): green, proteins upregulated (fold-regulation > 2); red, proteins downregulated (fold-regulation > -2); yellow, unaltered proteins; violet, A549-specific proteins.

**Fig 6:**
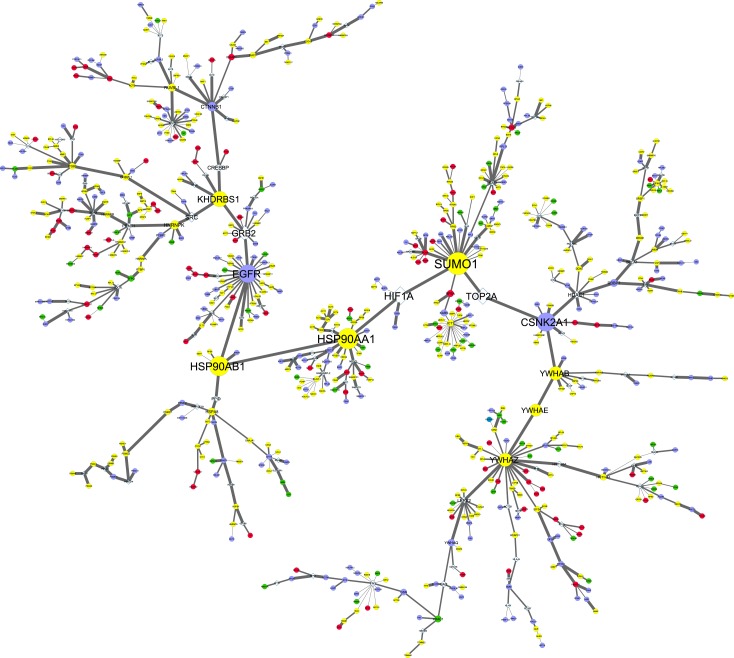
Integration of proteome and interactome data of the squamous carcinoma cell line. SteinerNet webserver was used to reveal hidden components in SW900 network by integrating the proteome (MS, fold-regulation) and the interactome data (HIPPIE, interaction scores). From the original network, 172 terminal nodes were excluded (21.8%) and 618 terminal nodes included (78.2%). Circular nodes denotes proteins obtained from MS, whereas diamond nodes are proteins obtained from HIPPIE database. Node and letter size are related to the betweeness centrality (high betweeness centrality represent important nodes in the network, also called bottlenecks) of the proteins and was calculated using the Cytoscape NetworkAnalyzer tool. Edge width shows the interaction score confidence. Node color is depicted as following (SW900 vs A549): green, proteins upregulated (fold-regulation > 2); red, proteins downregulated (fold-regulation > -2); yellow, unaltered proteins; violet, SW900-specific proteins.

In order to reveal the hidden components of the networks, the proteomics data was integrated with the interactome data. The web server, SteinerNet, was used to analyze the proteomic data by solving the prize-collecting Steiner tree (PCST) problem and to reconstruct a biologically relevant network composed of a subset of the altered/detected proteins (terminals) through other undetected proteins that were present in the tissue/cell line interactomes. The new networks confirm the importance of some of the hub-bottleneck proteins present in the global networks while also revealing new ones (Figs 5 and 6). In both networks the hub-bottlenecks SUMO1, YWHAZ and HSP90AA1 are still present, while SUMO2 (hub-nonbottleneck), HDAC1 (nonhub-bottleneck) and YWHAG (nonhub-nonbottleneck) does not seem to have such a preponderant role (Figs 5 and 6, and Table 2). The molecular chaperone (HSP90AA1), the signal transduction adaptor (YWHAZ) and the sumoylation protein (SUMO1) are highly important in both networks and it is not surprising not to be altered between the cell lines, since are all involved in a wide range of biological processes. Considering the hub and bottleneck proteins that in our study are cell line-specific, the FN1 (Fibronectin), RAN (a member RAS oncogene family) and TOP2A (Topoisomerase IIα) were obtained for the A549 cell line and CSNK2A1 (Casein kinase 2, α1) and EGFR were obtained for the SW900 cell line (Table 2). In spite of that, caution should be taken when analyzing proteomic data because an absence does not imply that they are not present. Fibronectin is an important protein for cell adhesion and its interaction with integrins has a role in cancer migration, invasion and metastasis. In NSCLC fibronectin has shown to have a role in proliferation, survival and differentiation through the activation of the PI3K/Akt/mTOR signaling pathway and the inhibition of the LKB1/AMPK signaling[64]. Our data shows that fibronectin is present in A549 as a hub-nonbottleneck protein but not in SW900 and this makes sense considering that the A549 cell line also harbors an inactivating mutation in LKB1/STK11 (homozygous for c.109C>T/p.Q37*) leading to an activation of the mTOR signaling[16]. RAN (Ras-related nuclear protein) is a member of the RAS oncogene family of GTPases and is upregulated in NSCLC cells[65]. It is required for NSCLC cell survival, invasion and epithelial to mesenchymal transition through the activation of the PI3K/Akt signaling pathway but not the Ras/Raf/MEK/ERK pathway[65]. RAN was present in A549 as a nonhub-bottleneck protein but not in SW900. The presence of fibronectin, RAN and the inactivation of LKB1 implies that the A549 cell line has the PI3K/Akt/mTOR route more activated than the SW900 cell line, which could indicate that inhibitors of this pathway may have limited effects in the SW900 cell line. Regarding TOP2A gene, it encodes for the protein topoisomerase IIα that is an essential nuclear enzyme for chromosome condensation in the cell cycle. Topoisomerase II inhibitors, such as anthracyclines (e.g. doxorubicin) and etoposide are amongst the most widely used anti‐cancer agents. These chemotherapeutic agents are commonly used in SCLC due to the high expression of TOP2A of these tumors when compared with NSCLC[66]. A study comparing the sensitivity of NSCLC cell lines to etoposide and tenoposide has shown that the SW900 cell line is more resistant than the A549 cell line[67]. This could be explained in our study by the presence of TOP2A in A549 as a nonhub-bottleneck protein but not in SW900. Our Western blot data shows that although not being absent in the squamous carcinoma cell line, topoisomerase IIα is >12 times more abundant in the adenocarcinoma cell line (Fig 4). Of special relevance for SW900 cell line, the EGFR and the epidermal growth factor receptor-bound protein 2 (GRB2), are both hub-bottlenecks. The EGFR receptor signaling is channeled through the PI3K/Akt and Ras/Raf/MEK/ERK pathways that are responsible for the normal regulation of essential cellular processes such as proliferation and apoptosis[68]. It is common that tumor cells could harbor EGFR mutations normally localized within the tyrosine kinase domain of the gene[68], however both A549 and SW900 cell lines have no mutations in this receptor. Regarding CSNK2A1 gene, it encodes the α subunit of casein kinase 2 (CK2) which is a ubiquitous serine/threonine protein kinase[69]. Casein kinase 2 plays important functions in cell growth, proliferation, apoptosis, differentiation and transformation being its activity increased in many types of tumors, including lung[70]. Casein kinase 2 was also present in the SW900 cell line as a nonhub-bottleneck protein but not in the A549 cell line. Considering the altered proteins between both cell lines, only the nonhub-bottleneck (ILF3, interleukin enhancer binding factor 3) and the hub-nonbottleneck (PCNA, proliferating cell nuclear antigen) arise, being the first overexpressed (3.5 fold) and the second one underexpressed (2.2 fold) in A549 when compared to SW900 (Table 2). The ILF3 gene, encodes two isoforms (NF110 and NF90) that together with ILF2/NF45 form heterodimeric complexes that regulate the transcription of several genes[71]. IFL3, is also an oncogene that is overexpressed in doxorubicin/cyclophosphamide-resistant breast tumors and in lung cancer development and progression[72, 73]. Moreover, it was recently shown that YM-155, a potent inhibitor of survivin/BIRC5 expression, targets ILF3/NF110[74]. Survivin, which is responsible for tumor progression and drug resistance in several types of cancer, is therefore modulated by ILF3 and could explain, at least in part, why ILF3 is also an oncogene. Our results show that the adenocarcinoma A549 cell line has 3.5 times more ILF3 expression than the squamous carcinoma SW900 cell line. This can be correlated with the high expression levels and the negative prognostic factor of survivin shown in adenocarcinomas of the lung[75, 76]. The PCNA gene encodes a 36 kDa protein that is highly expressed in proliferating cells and has an important role in cell cycle regulation, DNA replication and DNA repair[77, 78]. PCNA bound to chromatin helps to recruit several proteins involved in DNA synthesis and repair, DNA damage response and cell cycle control[79]. Given its role in cell proliferation, it is a widely used marker for cancer progression and patient prognosis in several types of cancer, however for NSCLC, studies have shown no correlation with patient survival[80, 81]. Not surprisingly, the PCNA gene is highly expressed in most NCSLC patients, although no difference was observed among the adenocarcinomas and the squamous carcinoma populations[82, 83]. However, a previous report using proteomic analysis to discover molecular targets and biomarkers in squamous carcinoma and adenocarcinoma patient samples has shown that PCNA is highly enriched in these subtypes when compared to normal samples and that the spectral count from the shotgun analysis in squamous carcinoma is higher than in adenocarcinoma[84]. Our data, shows that PCNA is more expressed (2.2-fold) in SW900 when compared with A549.

**Table 2:** Hub and bottlenecks present in the SteinerNet A549 and SW900 networks. Node color refers to the networks color code: green, proteins upregulated (fold-regulation > 2); red, proteins downregulated (fold-regulation > -2); yellow, unaltered proteins; violet, cell line-specific proteins obtained in the study; grey, proteins obtained from HIPPIE database. A threshold degree of >10 and betweenness centrality of >0.3 were used to retrieve the hubs and bottlenecks. Light green color in degree and betweenness centrality represents high values. Light orange color represents the nodes that are hub-bottlenecks.

A549 (adenocarcinoma)					SW900 (squamous carcinoma)				
Node	Degree	Betweenness Centrality	Hub (>10)	Bottleneck (>0.3)	Node	Degree	Betweenness Centrality	Hub (>10)	Bottleneck (>0.3)
ILF3	3	0.343	**-**	**+**	ILF3	3	0.025	**-**	**-**
PCNA	10	0.123	**+**	**-**	PCNA	8	0.028	**-**	**-**
FN1	18	0.069	**+**	**-**	FN1	12	0.058	**+**	**-**
RAN	7	0.421	**-**	**+**	RAN	2	0.003	**-**	**-**
TOP2A	2	0.481	**-**	**+**	TOP2A	2	0.454	**-**	**+**
HSP90AA1	10	0.375	**+**	**+**	HSP90AA1	11	0.570	**+**	**+**
HSP90AB1	6	0.212	**-**	**-**	HSP90AB1	6	0.535	**-**	**+**
ICT1	21	0.072	**+**	**-**	ICT1	22	0.075	**+**	**-**
SUMO1	15	0.558	**+**	**+**	SUMO1	18	0.621	**+**	**+**
SUMO2	10	0.027	**+**	**-**	SUMO2	6	0.020	**-**	**-**
YWHAB	7	0.339	**-**	**+**	YWHAB	8	0.365	**-**	**+**
YWHAE	2	0.295	**-**	**-**	YWHAE	2	0.328	**-**	**+**
YWHAZ	20	0.319	**+**	**+**	YWHAZ	22	0.361	**+**	**+**
KHDRBS1	5	0.069	**-**	**-**	KHDRBS1	5	0.421	**-**	**+**
CSNK2A1	8	0.722	**-**	**+**	CSNK2A1	8	0.509	**-**	**+**
EGFR	16	0.155	**+**	**-**	EGFR	23	0.492	**+**	**+**
ATF2	11	0.030	**+**	**-**	ATF2	6	0.017	**-**	**-**
GRB2	9	0.024	**-**	**-**	GRB2	10	0.413	**+**	**+**
HTT	11	0.045	**+**	**-**	HTT	9	0.022	**-**	**-**
LRRK2	11	0.033	**+**	**-**	LRRK2	12	0.133	**+**	**-**
MDM2	13	0.040	**+**	**-**	MDM2	13	0.080	**+**	**-**
MYC	12	0.215	**+**	**-**	MYC	17	0.056	**+**	**-**
PRMT1	5	0.332	**-**	**+**	PRMT1	6	0.117	**-**	**-**
RANGAP1	2	0.399	**-**	**+**	RANGAP1	*Not present in the Steiner network*			
XPO5	3	0.347	**-**	**+**	XPO5	*Not present in the Steiner network*			
HIF1A	*Not present in the Steiner network*				HIF1A	4	0.508	**-**	**+**

### Cell Lines Functional Characterization

**Fig 7:**
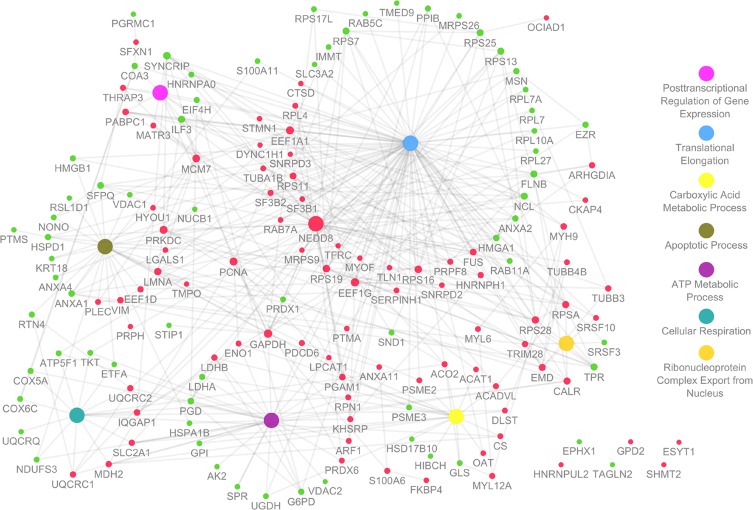
Functional network of altered proteins present in both cell lines. ClueGO plugin of Cytoscape was used to generate a functional network (biological process). Node size is related to the degree (high degree represent important nodes in the network, also known as hubs) of the proteins and was calculated using the Cytoscape NetworkAnalyzer tool. Proteins node color is depicted as following (A549 vs SW900): green, proteins upregulated (fold-regulation > 2); red, proteins downregulated (fold-regulation > -2). Biological process node color is represented on the right side of the image. On the right bottom side of the image are shown the genes that does not fit these biological processes and that do not have any interaction with the proteins that are altered.

To gain insight on the main functions connected to the altered protein expression observed between the cell lines, a functional network of molecular function was generated using the ClueGO plugin of Cytoscape (Fig 7). The differentially expressed proteins could be grouped in seven different molecular functional processes ranging from translational elongation, apoptosis and cellular respiration to name a few. Most of the proteins are connected to the translational elongation process, which is not surprising since high level of protein biosynthesis is required to cancer cell metabolism (Fig 7). Translation is regulated at the initiation and elongation step and is deregulated in cancer through several mechanisms[85]. The NEDD8 gene (neural precursor cell expressed, developmentally down-regulated 8), which is underexpressed in A549 (4.6 fold), is the major hub in this functional network and it is connected with one fourth of the proteins (41/159, 25.9%, Fig 7). These proteins are associated with all the functional processes retrieved. The ubiquitin-like protein NEDD8 is the master player in the neddylation process which is responsible for substrate conformational change, resulting in the repositioning of binding partners or the incompatibility to bind the usual partners[86]. NEDD8 is synthesized as a precursor that is processed by deneddylating enzymes (e.g. NEDP1 or UCLH3), in a C-terminal glycine residue which will serve as the binding site for target substrates. Similar to ubiquitination, the exposed residue is firstly adenylated by an activating (E1) enzyme (AppBp1/UBA3, or NAE) and transferred to the E1 cysteine side chain via a thiolester linkage. Activated NEDD8 is then transferred to a conjugating (E2) enzyme (UBC12 or UBE2F) forming another thiolester linkage. A ligase (E3) finally transfers NEDD8 to a substrate via the formation of an isopeptide bond[86]. The best-characterized NEDD8 substrates include the structurally related proteins cullins that function as molecular scaffolds of cullin-RING ligases (CRLs) being important for CRL-dependent ubiquitination. The NEDD8 control over the CRL ubiquitination system that is highly important in cell cycle progression and in cell growth and survival, implies that a dysregulation of normal NEDD8 processes is linked to cancer as well[86, 87]. Tumor growth inhibition, using MLN4924, a NAE inhibitor, was demonstrated with in colon cancer cell line (HCT-116) and in lung tumor xenografts[88].

### Integrative View

Lung cancer is a complex and heterogeneous entity therefore it is not surprising that the majority of NSCLCs contain a mixture of different cancer cell types. Tumor-derived cell lines in turn are selected *in vitro* and since they lack the tissue architecture, tumoral microenvironment and cell-cell communication of the tumor *in situ*, they represent an easier and very robust cancer model for pre-clinical studies. Several lines of research using gene expression data highlighted that cell lines have an upregulation of genes associated with proliferation, ribosomal activity, cellular energetics and cell cycle. On the other hand, there is a downregulation of genes associated with cell communication, adhesion and motility[89, 90]. In spite of that, genes implicated in the emergence and progression of cancer have similar expression patterns in cancer cell lines and tumors, which validate the usefulness of the cell lines as an *in vitro* model of the tumors. The A549 adenocarcinoma and the SW900 squamous carcinoma cell lines are among the most commonly studied in lung cancer research and are widely used in terms of basic mechanisms of lung cancer and as pre-clinical *in vitro* models for drug sensitivity and effectiveness[91–94]. Past studies have focused in the comparative characterization between lung tumors and the corresponding derived cell lines in terms of morphology, genotyping, gene expression and protein abundance[95–97]. In general, lung cancer cell lines are representative of the tissue from which they derive proving that are a suitable model for pre-clinical research[95–97]. In our study, the biological processes related to cellular energetics (cellular respiration, mitochondrion organization, ATP metabolism) and ribosomal activity (RNA processing and translation), which were present in both cell lines, could be associated with this *in vitro* phenotype, which would be specific of each cell line. On the other side, the cell line-specific proteins could hint new processes and therapeutic targets for future studies. The adenocarcinoma cell line was enriched in proteins related to cellular respiration, positive regulation of apoptosis, homeostasis, response to drug/hypoxia and oxidative stress, intracellular transport, nitrogen compound biosynthetic process and ubiquitination. In turn, the squamous carcinoma was enriched in proteins related to negative regulation of apoptosis, response to inorganic and organic substances, cytoskeleton organization and protein translation. Most of the proteins with different expression profiles between the cell lines in study are related to cancer transformation, proliferation, migration, invasion and metastasis (calgizzarin, Rab11a, Rab5c and SLC3A2 in adenocarcinoma cell line and matrin 3, stathmin, vimentin, calcyclin and galectin 1 in squamous carcinoma cell line). In turn, the analysis of the cell lines interactome has shown that most of the key proteins (hubs and bottlenecks) are shared between the cell lines with no expression alteration. Moreover, the presence of fibronectin, RAN and topoisomerase IIα in the adenocarcinoma cell line predicts better response for PI3K/Akt/mTOR inhibitors (e.g. rapamycin and rapalogs, and second generation ATP-competitive inhibitors) and topoisomerase IIα inhibitors (e.g. etoposide and doxorubicin). In turn, the presence of EGFR in the squamous carcinoma cell line might not confer any therapeutic advantage since this cell line has an activating mutation in K-RAS and the PI3K/Akt/mTOR route seems to be less active in this cell line. However, the presence of Ck2 in the squamous carcinoma cell line and its role in several tumorigenic processes might hint a sensitivity of this cell line for Ck2 inhibitors (e.g. K64, DRB and apigenin)[98, 99]. Additionally, the adenocarcinoma cell line has shown high comparative expression of ILF3, a protein that is commonly overexpressed in doxorubicin/cyclophosphamide-resistant tumors^63, 64^. The inhibition of the oncogenic protein ILF3 could be achieved through YM-155[74] and in our adenocarcinoma model this could sensitize the cells to other drugs and stop cell proliferation. In turn, the squamous carcinoma cell line showed high comparative expression of the PCNA protein, which is an important player in DNA replication and maintaining genome integrity. Although direct inhibition of PCNA has been difficult to achieve due to the lack of targetable sites, a new study has shown that targeting the tyrosine phosphorylation (Y211) of PCNA could inhibit cell proliferation in prostate cancer[79] and so it is feasible that the same could apply for squamous carcinoma cells. Considering the overall functional network, most of the altered proteins are related to the translational elongation process that is commonly dysregulated in cancer[85]. Another protein that was comparatively overexpressed in the squamous carcinoma cell line was the ubiquitin-like protein and master player of the neddylation process, NEDD8. A specific inhibitor of NAE activating E1 enzyme (MLN4924/Pevonedistat), which blocks the first NEDD8 adenylation step, was recently discovered and it is now in several phase I clinical trials for several types of cancer[100, 101]. This inhibitor induces autophagy, senescence and apoptosis[102] and a recent study has shown that the neddylation process is high in lung tumor samples when compared to adjacent normal tissue[103]. Besides this, MLN4924 was able to inhibit cell proliferation, migration and motility, and sensitize the lung cancer cells (adenocarcinoma A549 and H1299 cell lines and large cell carcinoma cell line H460) to cisplatin and carboplatin[103]. The comparative high overexpression of NEDD8 in the squamous carcinoma cell line hints that this effect could be even more pronounced in this subtype of NSCLC.

## Conclusions

This study highlights the major proteomic and functional differences between two of the most frequently used lung cancer *in vitro* models. In addition, several targeted therapies were emphasized that could benefit the adenocarcinoma and squamous carcinoma subtypes of NSCLC based on the specific targets found altered/present in each cell line. Further studies, aiming to elucidate the therapeutic potential of these targets will undoubtedly be of paramount importance.

## Supporting Information

S1 Fig Protein ratios (fold-regulation, A549 vs SW900) of the 496 proteins shared between both lung cancer cell lines. (TIF)

S2 Fig Protein-protein interaction network of the adenocarcinoma cell line. The protein-protein interaction network was obtained from HIPPIE and visualized in the Cytoscape software. Edges represent high confidence interactions. Circular nodes denotes proteins obtained from MS, whereas diamond nodes are proteins obtained from HIPPIE database. Node size is related to the betweeness centrality (high betweeness centrality represent important nodes in the network, also known as bottlenecks) of the proteins and was calculated using the NetworkAnalyzer tool. Node color is depicted as following (A549 vs SW900): green, proteins upregulated (fold-regulation > 2); red, proteins downregulated (fold-regulation > -2); yellow, unaltered proteins; violet, A549-specific proteins.
(TIF)

S3 Fig Protein-protein interaction network of the squamous carcinoma cell line. The protein-protein interaction network was obtained from HIPPIE and visualized in the Cytoscape software. Edges represent high confidence interactions. Circular nodes denotes proteins obtained from MS, whereas diamond nodes are proteins obtained from HIPPIE database. Node size is related to the betweeness centrality (high betweeness centrality represent important nodes in the network, also known as bottlenecks) of the proteins and was calculated using the NetworkAnalyzer tool. Node color is depicted as following (SW900 vs A549): green, proteins upregulated (fold-regulation > 2); red, proteins downregulated (fold-regulation > -2); yellow, unaltered proteins; violet, SW900-specific proteins.
(TIF)

S1 Table A549 and SW900 proteins obtained by MALDI-TOF/TOF. (XLSX)

S2 Table A549-specific genes mapped by DAVID and corresponding GOs terms (cellular component, biological process and molecular function) associated considering a p-value of 0.05. (XLSX)

S3 Table SW900-specific genes mapped by DAVID and corresponding GOs terms (cellular component, biological process and molecular function) associated considering a p-value of 0.05. (XLSX)

S4 Table A549 context relevant protein-protein interactions (PPIs) with medium-high confidence (score > 0.63) retrieved from HIPPIE database. (XLSX)

S5 Table SW900 context relevant protein-protein interactions (PPIs) with medium-high confidence (score > 0.63) retrieved from HIPPIE database. (XLSX)
