# Supplementary figures and images for: From Proteomic Analysis to Potential Therapeutic Targets: Functional Profile of Two Lung Cancer Cell Lines, A549 and SW900, Widely Studied in Pre-Clinical Research
Source: PLoS One. 2016 Nov 4;11(11):e0165973. doi: 10.1371/journal.pone.0165973 (PMC5096714; doi:10.1371/journal.pone.0165973)

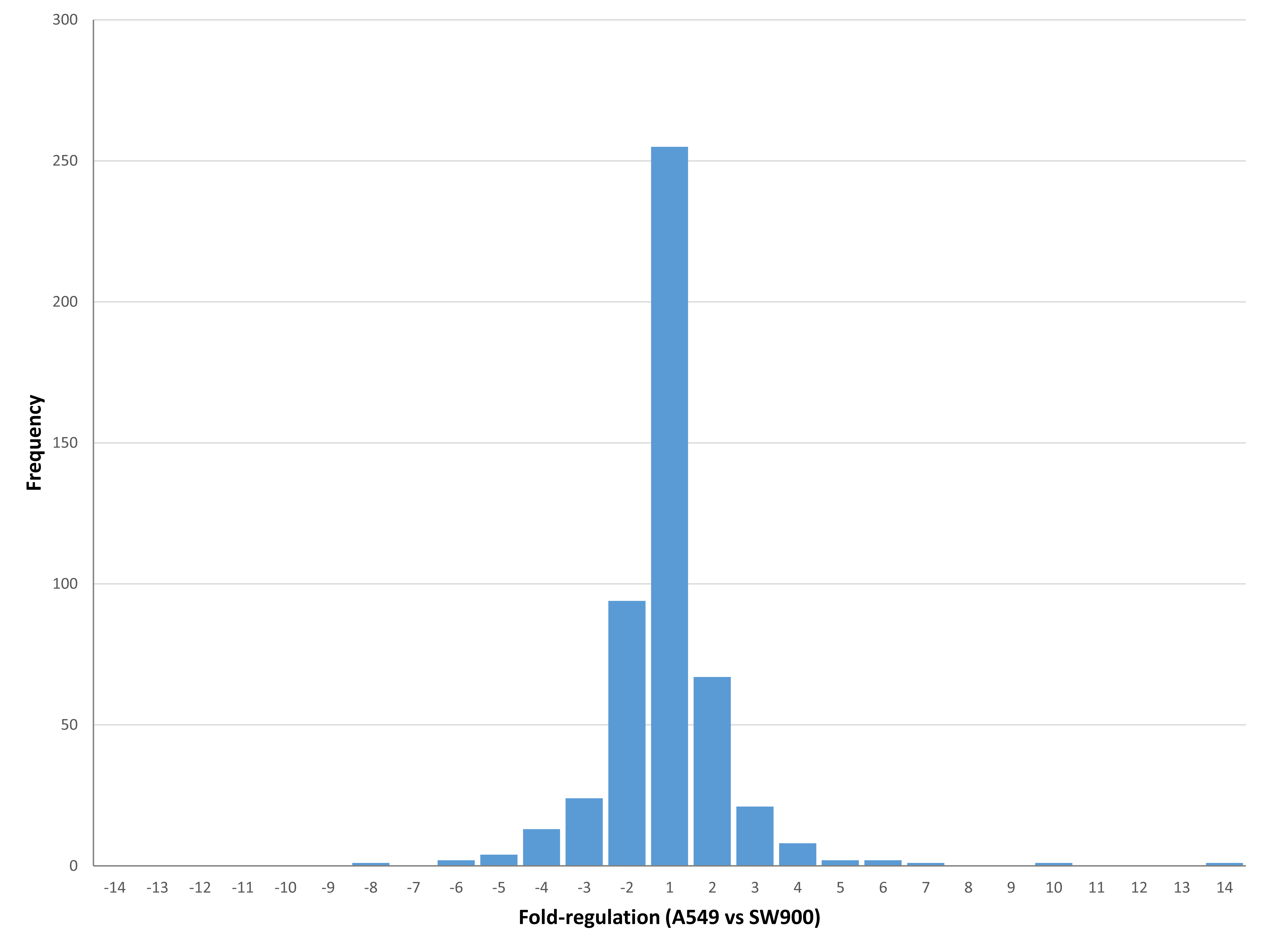

Supplement: S1 Fig — (TIF) [file pone.0165973.s001.tif]

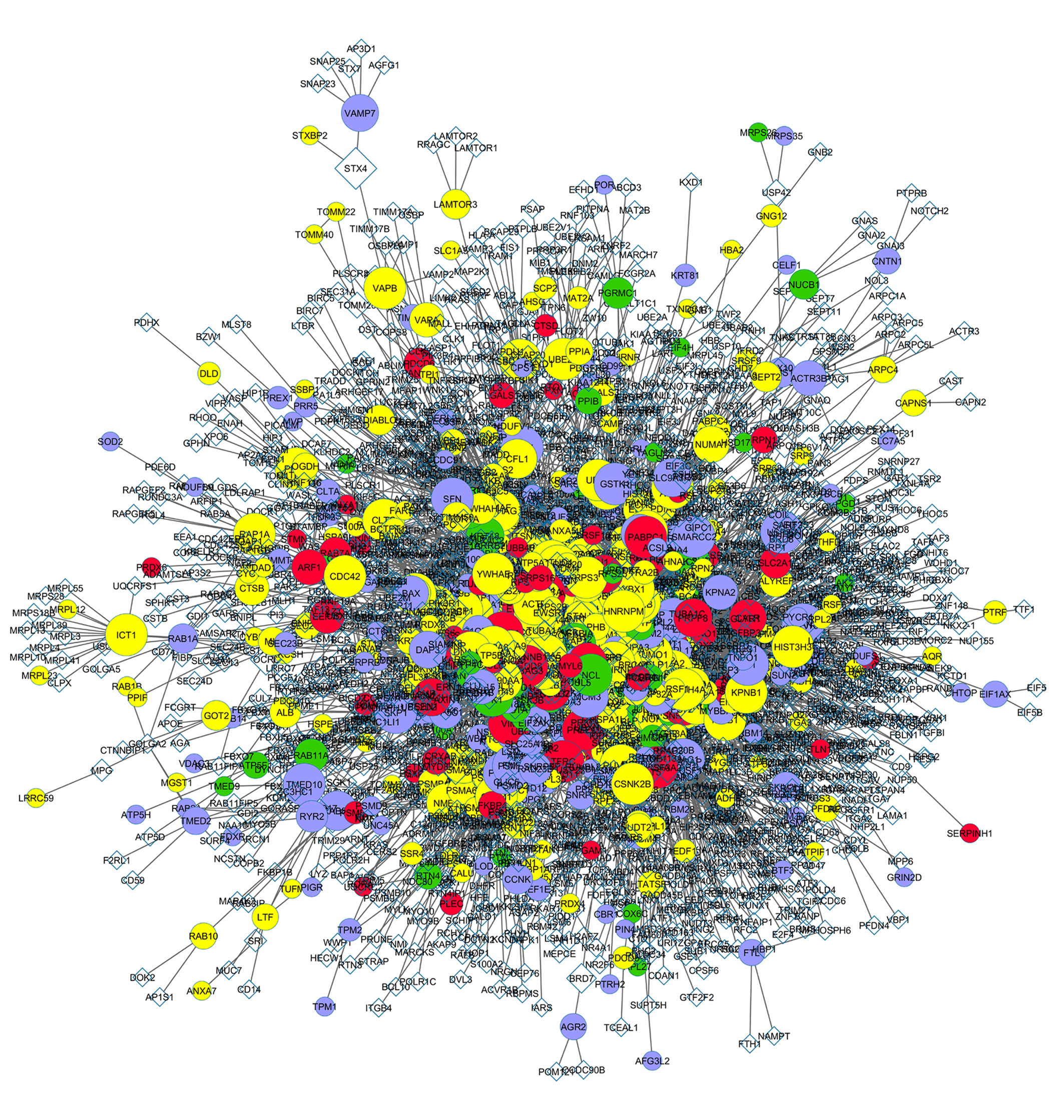

Supplement: S2 Fig — The protein-protein interaction network was obtained from HIPPIE and visualized in the Cytoscape software. Edges represent high confidence interactions. Circular nodes denotes proteins obtained from MS, whereas diamond nodes are proteins obtained from HIPPIE database. Node size is related to the betweeness centrality (high betweeness centrality represent important nodes in the network, also known as bottlenecks) of the proteins and was calculated using the NetworkAnalyzer tool. Node color is depicted as following (A549 vs SW900): green, proteins upregulated (fold-regulation > 2); red, proteins downregulated (fold-regulation > -2); yellow, unaltered proteins; violet, A549-specific proteins. (TIF) [file pone.0165973.s002.tif]

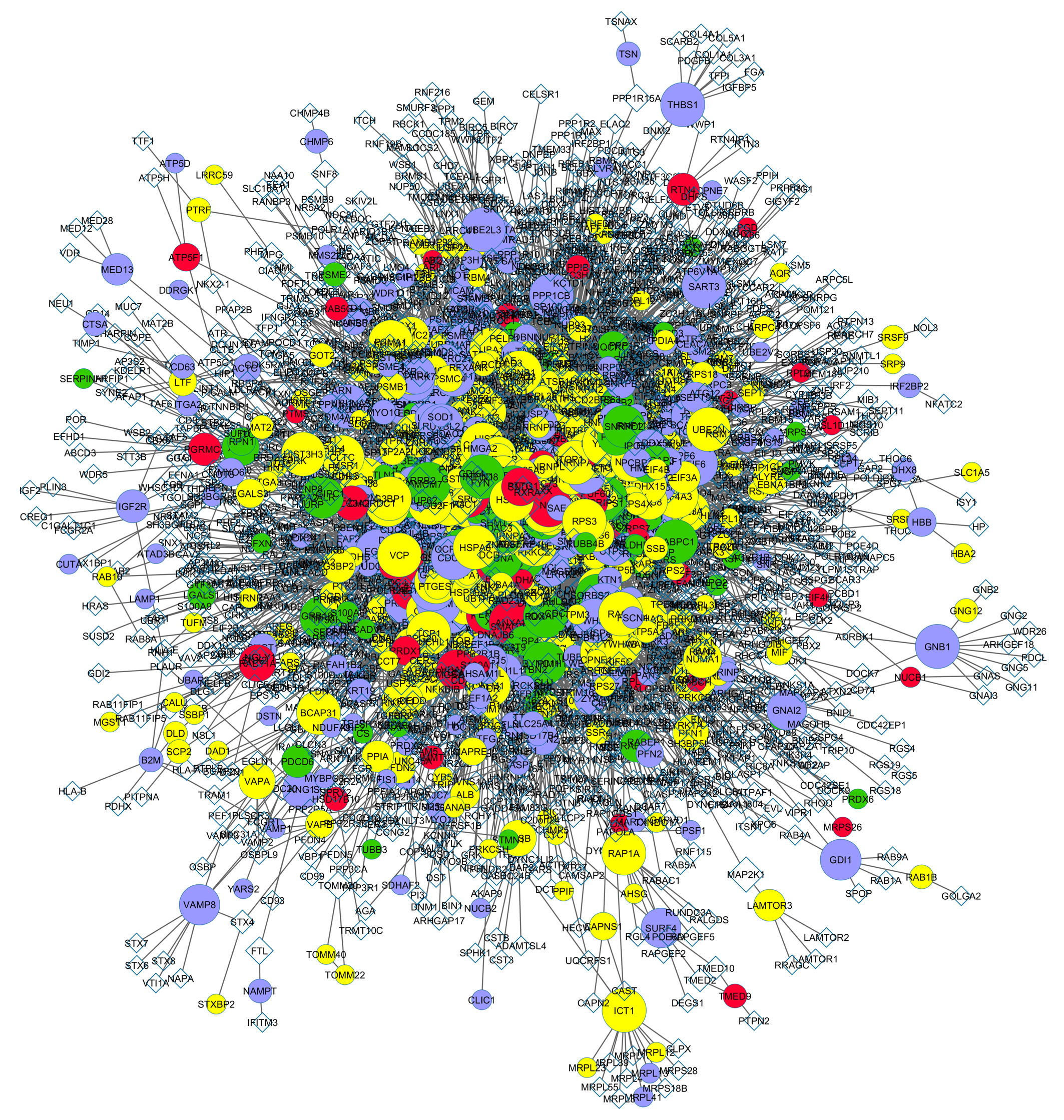

Supplement: S3 Fig — The protein-protein interaction network was obtained from HIPPIE and visualized in the Cytoscape software. Edges represent high confidence interactions. Circular nodes denotes proteins obtained from MS, whereas diamond nodes are proteins obtained from HIPPIE database. Node size is related to the betweeness centrality (high betweeness centrality represent important nodes in the network, also known as bottlenecks) of the proteins and was calculated using the NetworkAnalyzer tool. Node color is depicted as following (SW900 vs A549): green, proteins upregulated (fold-regulation > 2); red, proteins downregulated (fold-regulation > -2); yellow, unaltered proteins; violet, SW900-specific proteins. (TIF) [file pone.0165973.s003.tif]
